# FGF4 ameliorates the liver inflammation by reducing M1 macrophage polarization in experimental autoimmune hepatitis

**DOI:** 10.1186/s12967-024-05219-2

**Published:** 2024-08-02

**Authors:** Jing Lin, Hong-wei Lin, Yu-xing Wang, Yan Fang, Hui-mian Jiang, Ting Li, Jia Huang, Hua-dong Zhang, Da-zhi Chen, Yong-ping Chen

**Affiliations:** 1 Zhejiang Provincial Key Laboratory for Accurate Diagnosis and Treatment of Chronic Liver Diseases, The First Affiliated Hospital of Wenzhou Medical University, Hepatology Institute of Wenzhou Medical University, Wenzhou, 325000 Zhejiang China; 2 Department of Infectious Diseases, The First Affiliated Hospital of Soochow University, Suzhou, 215006 China; 3 Department of Clinical Medicine, Hangzhou Medical College, Hangzhou, 310053 China

**Keywords:** Fibroblast growth factor 4, Experimental autoimmune hepatitis, M1 macrophage polarization, PI3K/AKT signal pathway

## Abstract

**Background:**

The global prevalence of autoimmune hepatitis (AIH) is increasing due in part to the lack of effective pharmacotherapies. Growing evidence suggests that fibroblast growth factor 4 (FGF4) is crucial for diverse aspects of liver pathophysiology. However, its role in AIH remains unknown. Therefore, we investigated whether FGF4 can regulate M1 macrophage and thereby help treat liver inflammation in AIH.

**Methods:**

We obtained transcriptome-sequencing and clinical data for patients with AIH. Mice were injected with concanavalin A to induce experimental autoimmune hepatitis (EAH). The mechanism of action of FGF4 was examined using macrophage cell lines and bone marrow-derived macrophages.

**Results:**

We observed higher expression of markers associated with M1 and M2 macrophages in patients with AIH than that in individuals without AIH. EAH mice showed greater M1-macrophage polarization than control mice. The expression of M1-macrophage markers correlated positively with FGF4 expression. The loss of hepatic *Fgf4* aggravated hepatic inflammation by increasing the abundance of M1 macrophages. In contrast, the pharmacological administration of FGF4 mitigated hepatic inflammation by reducing M1-macrophage levels. The efficacy of FGF4 treatment was compromised following the in vivo clearance of macrophage populations. Mechanistically, FGF4 treatment activated the phosphatidylinositol 3-kinase (PI3K)–protein kinase B (AKT)-signal pathway in macrophages, which led to reduced M1 macrophages and hepatic inflammation.

**Conclusion:**

We identified FGF4 as a novel M1/M2 macrophage-phenotype regulator that acts through the PI3K–AKT-signaling pathway, suggesting that FGF4 may represent a novel target for treating inflammation in patients with AIH.

**Supplementary Information:**

The online version contains supplementary material available at 10.1186/s12967-024-05219-2.

## Introduction

Autoimmune hepatitis (AIH) represents an important global health concern, affecting both pediatric and adult populations. AIH manifests with chronic liver inflammation characterized by interface hepatitis, hypergammaglobulinemia, and the production of autoantibodies [1]. The global incidence and prevalence of AIH were found to be 1.28/100,000-person years and 15.65 cases/100,000 people, respectively [2]. Despite its low frequency, AIH imposes clinical burdens that exceed expectations based on population incidences and prevalences. Challenges in diagnosing and treating AIH persist, with approximately one-third of patients presenting with cirrhosis, one-fifth experiencing relapses, and 30–50% developing cirrhosis despite treatment [3]. The current management of AIH predominantly involves corticosteroids (primarily prednisolone), either as monotherapy or in combination with azathioprine. Additionally, second-line immunosuppressants are employed, which include mycophenolate mofetil, cyclosporine, and tacrolimus. Notably, these treatments (especially corticosteroids) are associated with significant adverse effects such as elevated blood sugar, osteoporosis, weight gain, sleep and mood disturbances, and increased susceptibilities to infections [4–6]. Given these challenges and associated risks, an imperative need exists for identifying novel and effective treatment targets for AIH. Conducting innovative pharmacological research is paramount for advancing the understanding and improving the therapeutic landscape of this complex liver disease.

The etiology and pathophysiology of AIH remain elusive. The pivotal role of immune-mediated macrophage polarization in driving inflammatory damage is a key aspect of AIH development [7–9]. Two main types of macrophages have been reported, namely conventionally activated (M1 type) and alternatively activated (M2 type) macrophages. M1 macrophages are predominantly triggered by lipopolysaccharide (LPS) and interferon-γ (IFN-γ) and are considered pro-inflammatory due to their ability to produce a plethora of pro-inflammatory cytokines such as interleukin (IL)-1β, IL-6, nitric oxide synthase 2 (NOS2), and tumor necrosis factor-α (TNF-α) [10]. In contrast, M2 macrophages are activated by IL-4 and IL-13 and are deemed anti-inflammatory because they primarily generate factors such as IL-10, transforming growth factor-β, and arginase 1 (ARG1) [11]. M1 macrophages function as antigen-presenting cells and possess pro-inflammatory, microbe-scavenging, and anti-tumor properties. Conversely, M2 macrophages help in reducing inflammatory responses, clearing debris and apoptotic cells, promoting tissue repair and wound healing, and enhancing immunological control [12]. The intricate interplay between these macrophage subpopulations underscores their significance in the immune-mediated mechanisms associated with AIH.

The regulatory mechanisms governing macrophages in AIH remain unclear, prompting us to explore the relevance of fibroblast growth factors (FGFs). A growing body of evidence indicates that various FGFs play key roles in diverse aspects of liver pathophysiology [13, 14]. Notably, pharmacological intervention with FGF4 can suppress adipose macrophage infiltration and inflammation, suggesting its therapeutic potential against AIH, an inflammatory condition characterized by macrophage activation [15]. However, the long-term use of FGF4, a known mitogen, raises concerns about an elevated risk of tumor development, which has limited its clinical applicability. In light of this concern, our research has identified distinct thresholds in the stability and activation of fibroblast growth factor receptor (FGFR) dimers, enabling us to design a recombinant, non-mitogenic analog of FGF4 [16]. This analog features an N-terminal truncation of residues Ala67–Leu206, which reduces its capacity to dimerize and activate heparan sulfate-assisted FGFRs. As anticipated, the modified FGF4 retains full activity when compared with that of wild-type FGF4, although its mitogenic activity is eliminated [17]. This innovative approach was aimed to harness the therapeutic potential of FGF4 in AIH while mitigating the associated risk of tumorigenesis, presenting a promising avenue for further exploration and clinical applications.

Although some findings have shown that FGF4 participates in inflammatory liver diseases, its association with AIH related to macrophage-phenotype switching has not been explored. Therefore, we sought to further determine the role and mechanism of FGF4 in a murine model of concanavalin A (ConA)-induced AIH, using hepatocyte-specific *Fgf4* knockout mice (*Fgf4*^−/−)^ and *Fgf4*-floxed mice (*Fgf4*^fl/fl^). In addition, wild-type (WT) mice were administered FGF4 or saline to investigate the therapeutic potential of FGF4 against AIH.

## Materials and methods

The descriptions of the procedures used for histological analysis, biochemical analysis, enzyme-linked immunosorbent assays (ELISAs), quantitative real-time PCR (qRT-PCR), western blotting (WB), immunofluorescence (IF) staining, immunohistochemistry (IHC), terminal deoxynucleotidyl transferase-mediated dUTP nick end labeling (TUNEL), and cytometric bead arrays (CBAs) are presented in the Additional file 1: Methods section.

### Expression and purification of human FGF4

A complementary DNA fragment encoding N-terminally truncated human FGF4 (lacking residues Ala67–Leu206) was cloned into the bacterial expression vector pET-15b and transformed into Escherichia coli BL21 cells. After a 2 h incubation at 37 °C with 1 mM isopropyl β-d-1-thiogalactopyranoside, the transformed cells were harvested and lysed using a high-volume homogenizer (Emulsiflex-C3, Avestin, Inc., Ontario, Canada). Subsequently, FGF4 was purified from the lysate supernatant through sequential heparin-affinity chromatography and size-exclusion chromatography using a Superdex-100 column (GE Healthcare, Piscataway, NJ, USA). This purification process enabled isolation of the N-terminally truncated human FGF4 protein for downstream analyses.

### Animal models

Hepatocyte-specific *Fgf4*-knockout (*Fgf4*^−/−^) mice were generated by crossing *Fgf4*-floxed (*Fgf4*^fl/fl^) mice with albumin–Cre recombinase-transgenic mice with a C57BL/6 J (wild-type, WT) background. In the EAH group, 8-week-old male WT, *Fgf4*^−/−^, and *Fgf4*^fl/fl^ mice were intravenously injected with ConA (Sigma-Aldrich, St. Louis, MO, USA; 15 mg/kg body weight) to induce EAH and then euthanized at the identified optimal time. In the treated group, 8-week-old male mice were treated with ConA (15 mg/kg body weight), followed by intraperitoneal injections of FGF4 (1.5 mg/kg body weight) or phosphate-buffered saline (PBS). Each treatment was repeated once after 6 h, and the animals were euthanized at the optimal time. Macrophage depletion was induced by intravenous injection of 200 μL clodronate liposomes or control liposomes (5 mg/mL, Liposoma) 24 h before ConA administration. In the intervention group, mice were pretreated with the phosphatidylinositol 3-kinase (PI3K) inhibitor, LY294002 (30 mg/kg, intraperitoneal injection, Sigma-Aldrich) for 30 min before ConA injection, following the same procedures as described for the treatment model. Each experiment involved four to six mice per group. The care of the mice adhered to National Institutes of Health guidelines. The sequences of the genotyping primers are provided in Additional file 1: Table S1.

### Human subjects

The study involved 21 human blood samples obtained from the First Affiliated Hospital of Wenzhou Medical University. Patients diagnosed with AIH (n = 10) met the revised scoring system for AIH diagnosis (1999) [18]. We found no evidence of overlapping features with primary biliary cholangitis or primary sclerosing cholangitis or evidence of previous immunosuppressive therapy [19]. Healthy volunteers, meeting the inclusion criterion (age ≥ 18 years; n = 11) provided additional blood samples. Normal liver-tissue specimens were sourced from individuals undergoing partial hepatectomy at the First Affiliated Hospital of Wenzhou Medical University due to hepatic hemangioma (n = 8), with an age criterion of ≥ 18 years. Our exclusion criteria ensured the absence of liver damage caused by factors such as viral infection, toxin exposure, medications, autoimmune diseases, or other unrelated causes [20]. The normal liver tissues procured were situated at least 3 cm away from the lesion and were not affected by the lesion invasion. Liver tissues from individuals diagnosed with AIH (n = 10) were collected from subjects aged ≥ 18 who met the established diagnostic criteria for AIH [18].

### Primary culture of mouse liver macrophages and hepatocytes

Primary liver macrophages and hepatocytes were isolated from C57BL/6 J male mice using established protocols [21, 22].

### Isolation bone marrow-derived macrophages (BMDMs)

BMDMs were isolated from 8–10-week-old, male C57BL/6 J mice, following previously established procedures [23].

### Co-cultures

Primary hepatocytes were co-cultured with BMDMs in cell-culture chambers, and a similar approach was employed to establish co-cultures with murine RAW264.7 (RAW) macrophages and alpha mouse liver 12 (AML12) cells. The cell densities were adjusted to a 3:1 ratio (primary hepatocytes: BMDM or AML12 cells: RAW cells). The primary hepatocytes and AML12 cells were plated in separate six-well plates, after which BMDM or RAW264.7 cells were added to the culture chamber, and the co-cultures were incubated for 6 h in an atmosphere containing 5% CO_2_. Next, the culture chambers were then treated with LPS (0–10 μg/mL, Sigma-Aldrich) and further incubated for 24 h with 5% CO_2_. In some experiments, the macrophages were pretreated with LY294002 (25 µM) 1 h before LPS stimulation at the most appropriate concentration. The cells were pretreated with FGF4 (0–10 µg/mL) for 30 min in experiments involving LPS. Following optimization, the most suitable FGF4 concentration was selected for subsequent experiments. The co-culture experiments were conducted three times.

FGF4 expression was knocked down in AML12 cells by transfecting them with an *Fgf4*-specific small-interfering RNA (siRNA) using Lipofectamine 2000 Reagent (Thermo Fisher) as per the manufacturer’s instructions. AML12 cells were also transfected with a non-targeting siRNA as a negative control. The sequences of the *Fgf4*-specific and control siRNAs are shown in Additional file 1: Table S4 (GenePharma, Shanghai, China).

### Flow cytometry

Liver cells were prepared as described in the previous section. The isolated cells were incubated with Fc-Block (BioLegend, CA, USA), followed by fluorochrome-conjugated antibodies (Additional file 1: Table S3). Flow-cytometric analysis was conducted using a FACSAria II instrument (BD Bioscience), and the obtained data were analyzed using FlowJo software (Tree Star, Ashland, OR, USA). Liver macrophages were defined as CD45 + FVS510 − CD11b + F4/80 + cells. Subsequently, M1 and M2 macrophages were identified as CD86 + and CD206 + cells, respectively.

### RNA-sequencing (RNA-seq) analysis

Liver tissues were harvested from three *Fgf4*^fl/fl^ EAH-model mice and three *Fgf4*^−/−^ EAH-model mice for RNA-seq analysis. Liver tissues were also harvested from four WT EAH-model mice and four WT EAH-model mice treated with FGF4 for RNA-seq. Paired-end RNA-seq libraries were processed using Illumina HiSeq Xten and NovaSeq 6000 sequencers (2 × 150 base pair read lengths) by LC-Bio Technologies Co., Ltd. (Hangzhou, China). Differentially expressed mRNAs were identified with a log_2_ (fold-change) > 1 or <  − 1, and statistical significance (*p* < 0.05) was calculated using the package edgeR of R software (version 3.6.3; R Foundation for Statistical Computing, Vienna, Austria). The results were visualized using R. Kyoto Encyclopedia of Genes and Genomes (KEGG) pathway-enrichment analysis was conducted using the Cluster Profiler package of R. A pathway was considered significantly enriched if it had a *p*-value of < 0.05, a normalized enrichment score of > 1, and a false-discovery rate of < 0.25.

### Analysis of immune cell infiltration

The mRNA-expression profile data for accession number GSE206364 were retrieved from the Gene Expression Omnibus Database (https://www.ncbi.nlm.nih.gov/geo/). The dataset includes sequencing data for nine normal liver tissue samples and five liver tissue samples from patients with AIH. ImmuCellAI (http://bioinfo.life.hust.edu.cn/ImmuCellAI/#!/) was employed to assess immune-cell infiltration in AIH compared to normal control samples. ImmuCellAI utilizes single-sample gene set-enrichment analysis to predict the abundances of 24 different immune-cell types in samples. This algorithm scores immune infiltration based on the expression levels of characteristic genes for each immune cell.

### Statistical analysis

The data were expressed as the mean ± standard deviation. The statistical significances of differences among multiple groups were assessed using one-way analysis of variance (ANOVA), and t-tests were applied for paired samples. GraphPad Prism 9.0 (GraphPad Software Inc.) was utilized for statistical analysis. All differences were deemed statistically significant at *p* < 0.05.

## Results

### Increased M1 and M2 macrophages in patients with AIH

**Fig. 1:**
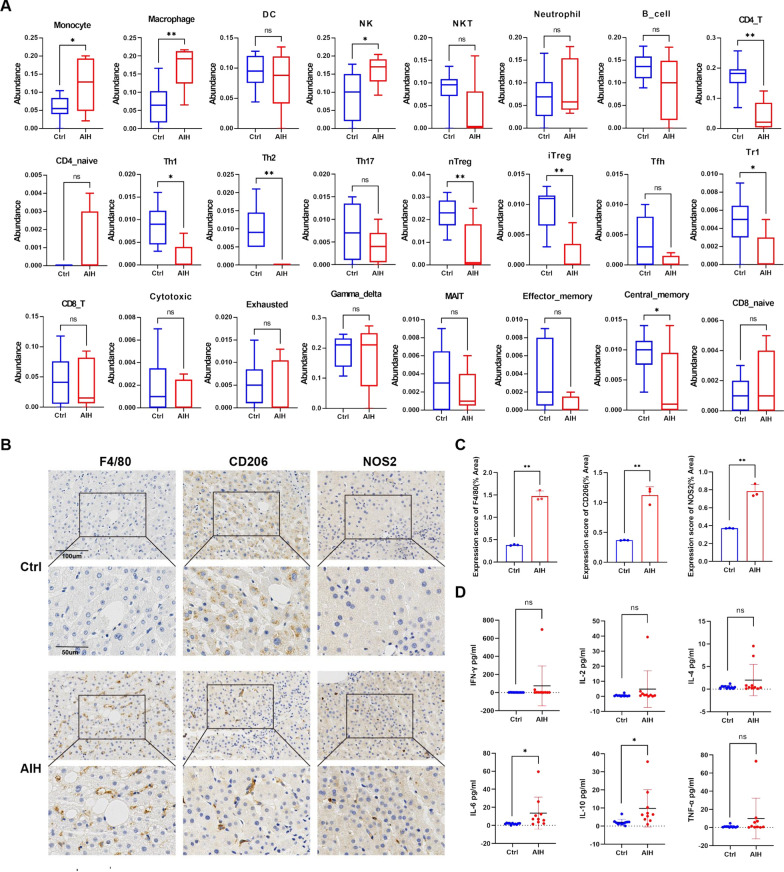
Increased M1 and M2 macrophages in patients with AIH. **A** Differentially infiltrating immune cells between patients with AIH and healthy controls based on information in the GSE206364 dataset. **B** F4/80, NOS2, and CD206 IHC staining for patients with AIH and normal controls. Scale bars, 100 μm or 50 μm. **C** Quantitative representation of the IHC-staining results. **D** Differentially secreted cytokines from M1 and M2 macrophages between the patients with AIH and the healthy controls. Statistical comparisons were made by performing the two-tailed, unpaired Student’s t-test; *p < 0.05, **p < 0.01, ns, not significant

The abundances of 24 different types of immune cells in five AIH liver samples and nine control liver samples were predicted using ImmuCellAI. Our immune-cell infiltration analysis demonstrated substantially higher monocyte, macrophage, and natural killer cell infiltration in liver tissues from patients with AIH than in those from healthy individuals. Conversely, lower infiltration of CD4 + T cells, Th1 cells, Th2 cells, nTreg cells, iTreg cells, Tr1 cells, and central memory cells was observed in the AIH samples. A particularly noteworthy observation was the marked elevation of macrophages, which captured our interest (Fig. 1A). Additionally, IHC staining of liver sections revealed that patients with AIH had significantly more F4/80 + macrophages, NOS2 + M1 macrophages, and CD206 + M2 macrophages than healthy controls (Fig. 1B, C). The AIH group also showed considerably higher levels of IL6 (13.64 ± 16.74 pg/mL) and IL10 (9.78 ± 9.79 pg/mL) (Fig. 1D).

### M1-macrophage abundances correlated positively with FGF4 levels in EAH mice

**Fig. 2:**
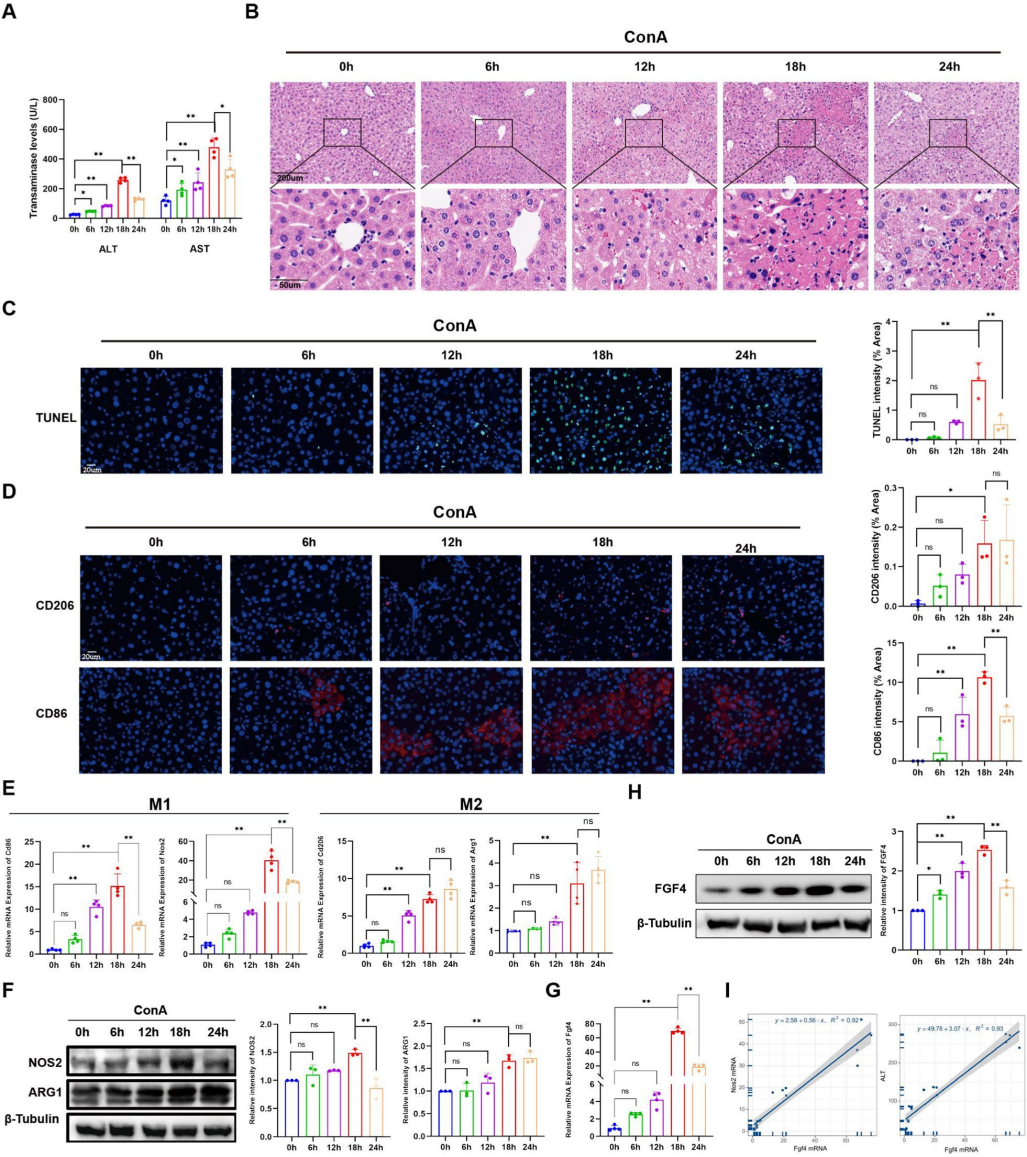
Positive correlation between M1-macrophage and FGF4 levels in a mouse model of AIH. **A** Serum ALT and AST levels at different time points (0–24 h) after ConA stimulation. **B** Representative images of H&E-stained liver tissue sections at different times post-stimulation. Scale bars, 200 μm or 50 μm. **C** Representative TUNEL staining of liver tissues at different timepoints. Scale bar, 20 μm. **D** Representative immunofluorescence images showing CD86 and CD206 expression in liver sections at different timepoints along with their corresponding quantitative results. Scale bar, 20 μm. **E** Changes in the relative hepatic mRNA-expression levels of M1-macrophage marker genes (*Nos2* and *Cd86*) and M2 macrophage marker genes (*Arg1* and *Cd206*) at different timepoints. **F** WB analyses showing changes in NOS2 and ARG1 expression in total liver lysates at different timepoints, along with their quantitative results. **G** Changes in the relative mRNA-expression levels of *Fgf4* in the liver at different timepoints. **H** WB analyses of changes in FGF4 expression in total liver lysates at different timepoints, with quantitative results. **I** The mRNA-expression levels of *Fgf4* correlation positively with the mRNA-expression levels of *Nos2* and serum ALT. The WB data were quantified using ImageJ software. n = 3–6 mice/group. Statistical comparisons were made using one-way ANOVA with Tukey’s post-hoc test; *p < 0.05, **p < 0.01, ns, not significant

EAH was induced in mice with ConA injection using various treatment times. The resulting liver necrosis was most severe at 18 h post-injection, when the greatest transaminase levels were found (Fig. 2A) and significant lymphocyte and plasma cell infiltration occurred, based on hematoxylin and eosin (H&E) staining (Fig. 2B). TUNEL staining also revealed that apoptosis was most evident at 18 h with the EAH model (Fig. 2C). M1 Macrophage followed a similar pattern. The levels of M1 macrophage markers (NOS2 and CD86) peaked at 18 h and then decreased, whereas the M2 macrophage markers (ARG1 and CD206) considerably increased at 18 h (Fig. 2D–F).

The FGF4 protein functions in a paracrine manner. Previous findings demonstrated that the liver is primarily responsible for FGF4 synthesis and secretion under both healthy and pathological settings [14]. The mRNA- and protein-expression levels of FGF4 peaked around 18 h after ConA injection and then decreased; this change pattern is comparable with the changing trend in M1 macrophage abundances (Fig. 2G, H). Furthermore, FGF4 expression correlated positively with M1 macrophage abundances and the levels of transaminases (Fig. 2I).

### Hepatic-specific deletion of *Fgf4* aggravated liver inflammation by increasing M1 macrophages in EAH mice

**Fig. 3:**
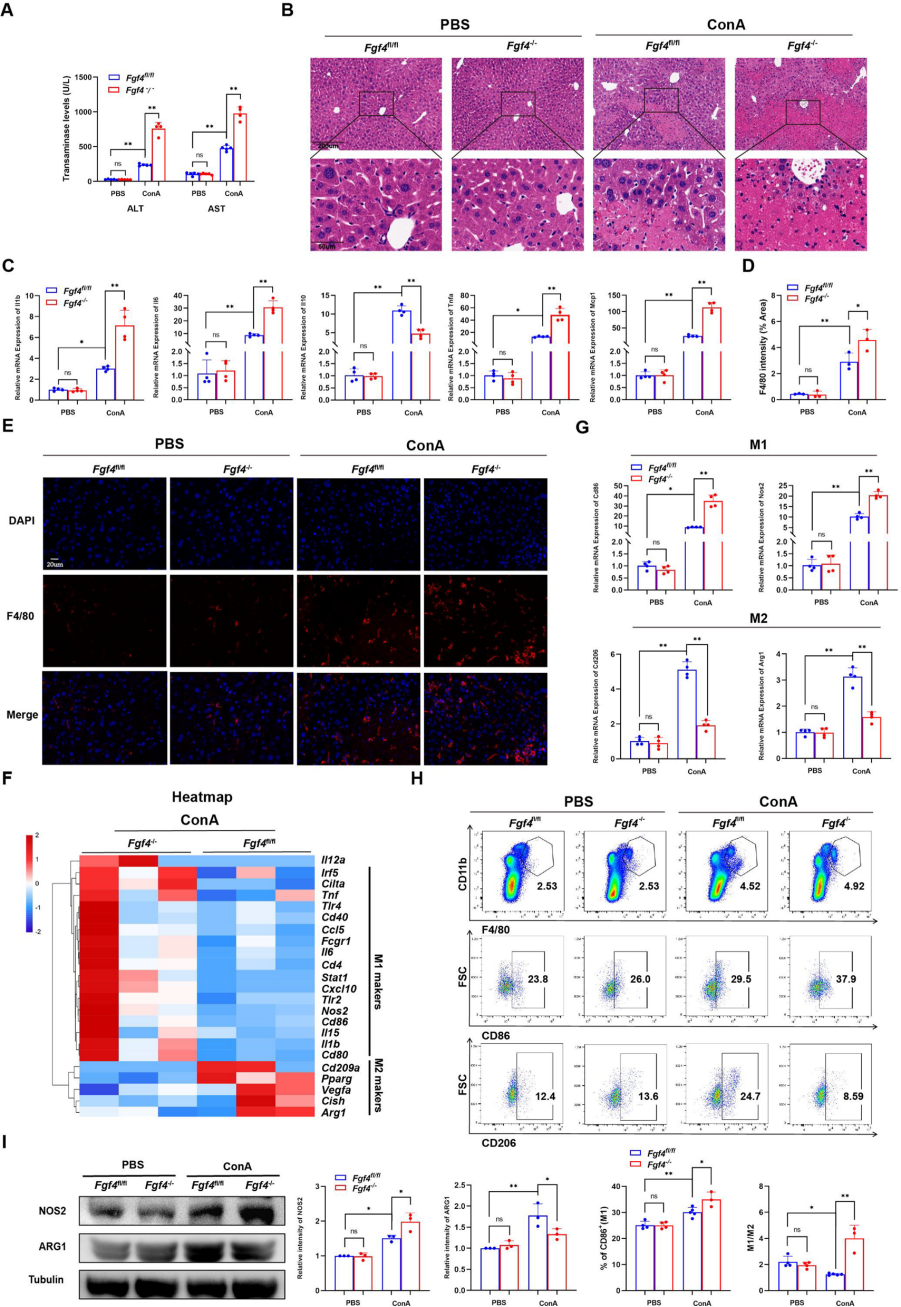
Liver-specific deletion of *Fgf4* aggravated liver inflammation by increasing M1 macrophage levels in EAH mice. **A** Serum ALT- and AST-activity levels of *Fgf4*^fl/fl^ and *Fgf4*^*−/−*^ mice in EAH and control groups. **B** Representative images of liver sections from EAH or control *Fgf4*^fl/fl^ and *Fgf4*^*−/−*^ mice stained with H&E. Scale bars, 200 μm or 50 μm. **C** Changes in relative mRNA-expression levels of inflammatory factors (*Il1b*, *Il6,* *Tnfa*, *Il10*, and *Mcp1*) in the livers of EAH mice or the control *Fgf4*^fl/fl^ and *Fgf4*^*−/−*^ mice. **D** The quantitative results for F4/80 expression in liver sections from EAH mice or the control *Fgf4*^fl/fl^ and *Fgf4*^*−/−*^ mice. **E** Representative immunofluorescence images of F4/80 expression in liver sections from EAH mice or the control *Fgf4*^fl/fl^ and *Fgf4*^*−/−*^ mice. Scale bar, 20 μm. **F** Heatmap of representative differentially expressed genes related to M1-macrophage and M2-macrophage marker genes in livers of *Fgf4*^fl/fl^ EAH mice and *Fgf4*^*−/−*^ EAH mice, based on RNA-seq data. **G** Changes in relative mRNA-expression levels of genes related to M1-macrophage markers (*Nos2* and *Cd86*) and M2-macrophage markers (*Arg1* and *Cd206*) in the livers of EAH mice or the control *Fgf4*^fl/fl^ and *Fgf4*^*−/−*^ mice. **H** Flow-cytometric analysis of changes in liver macrophages (F4/80 + CD11b + cells), M1 macrophages (CD86 + cells), and M2 macrophages (CD206 + cells), along with quantitation of the results. **I** WB analyses of changes in NOS2 and ARG1 expression in total liver lysates from EAH mice or the control *Fgf4*^fl/fl^ and *Fgf4*^*−/−*^ mice, with quantitation of the results. The WB data were quantified using ImageJ software. n = 3–6 mice/group. Statistical comparisons were made using one-way ANOVA with Tukey’s post-hoc test; *p < 0.05, **p < 0.01, ns, not significant

We employed *Fgf4* liver-specific knockout mice (*Fgf4*^*−/−*^) and *Fgf4*^*fl/fl*^ mice with the *Fgf4* gene intact as a control group. Mice injected with ConA were employed as the experimental group. The *Fgf4*^*−/−*^ EAH group exhibited higher alanine aminotransferase (ALT) and aspartate aminotransferase (AST) levels than the *Fgf4*^*fl/fl*^ EAH group (Fig. 3A) and regions with greater hepatocyte necrosis (Fig. 3B). The mRNA-expression levels of monocyte chemoattractant protein-1 (*Mcp1*)*, Il1β, Tnfα*, and *Il6* (inflammatory cytokines) were higher in *Fgf4*^*−/−*^ EAH mice than those in EAH *Fgf4*^*fl/fl*^ mice, although *Il10* (an anti-inflammatory cytokine) was expressed at lower levels (Fig. 3C). Moreover, the number of F4/80 + liver cells was significantly higher in *Fgf4*^*−/−*^ EAH mice than that in *Fgf4*^fl/fl^ EAH mice (Fig. 3D, E). RNA-Seq revealed that the expression levels of M1 macrophage-marker genes rose in the *Fgf4*^−/−^ EAH group and those of M2-macrophage marker genes decreased (Fig. 3F). Various tests were performed to confirm the RNA-Seq findings. The higher mRNA-expression levels of two M1-macrophage marker genes (*Nos2* and *Cd86*) and lower mRNA-expression level of two M2-macrophage marker gene (*Arg1* and *Cd206*) in the livers of *Fgf4*^*−/−*^ EAH mice (Fig. 3G) were confirmed using qRT-PCR. Flow-cytometric analysis revealed that *Fgf4*^*−/−*^ EAH mice had higher M1 macrophage abundances than *Fgf4*^*fl/fl*^ EAH mice (Fig. 3H). The protein-expression level of NOS2, an M1-macrophage marker, was significantly upregulated. Conversely, the protein-expression of ARG1, an M2-macrophage marker, was significantly downregulated in *Fgf4*^*−/−*^ EAH mice (Fig. 3I).

### FGF4 treatment attenuated liver inflammation by reducing M1 macrophage abundances in EAH mice

**Fig. 4:**
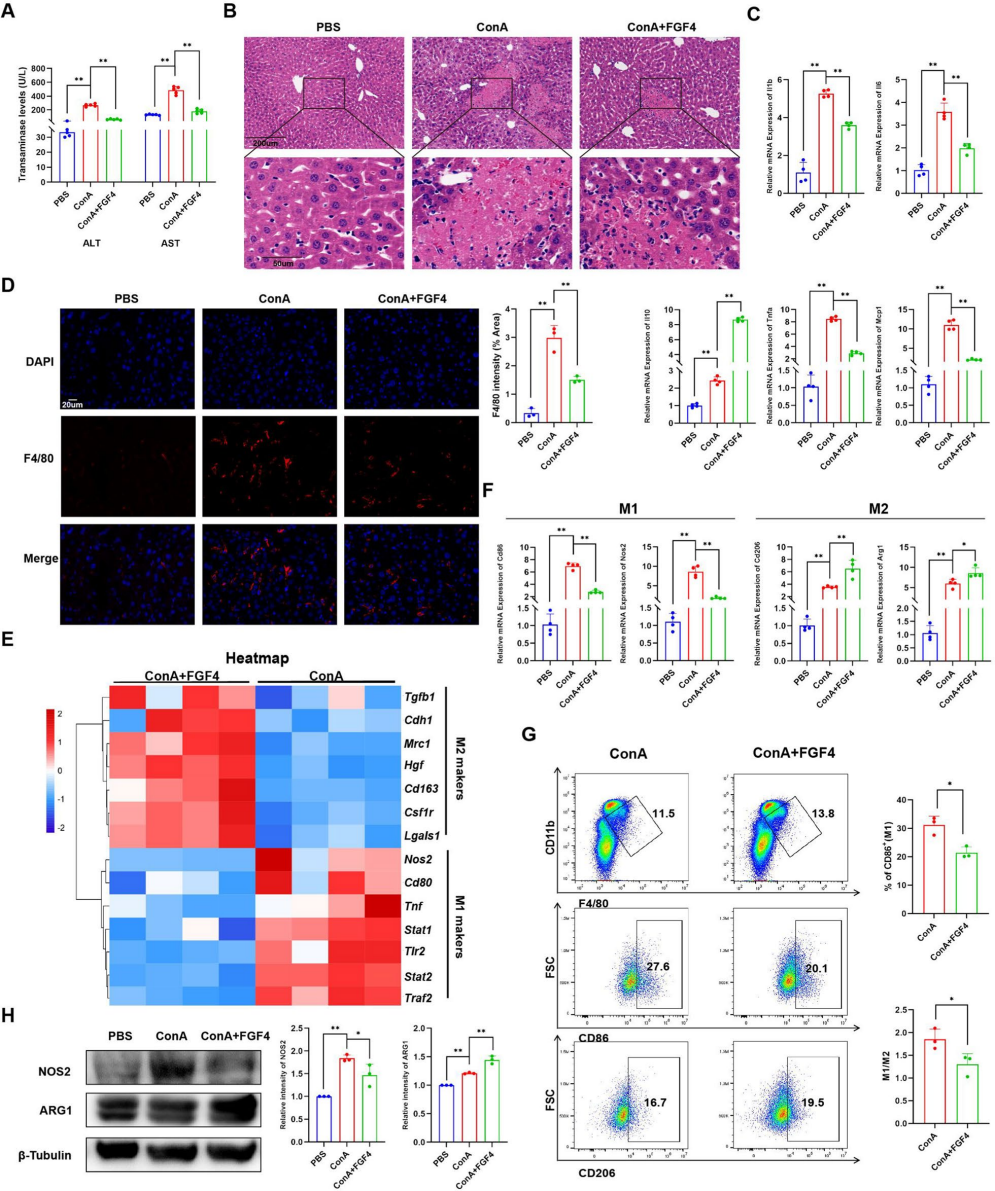
FGF4 treatment attenuated liver inflammation by reducing M1 macrophage abundances in EAH mice. **A** Serum ALT- and AST-activity levels in the control, EAH, and EAH + FGF4 groups. **B** Representative H&E-staining images of liver sections from mice in the control, EAH, and EAH + FGF4 groups. Scale bars, 200 μm or 50 μm. **C** Changes in the relative mRNA-expression levels of inflammatory factors (*Il1b*, *Il6*, *Tnfa*, *Il10* and *Mcp1*) in livers from the control, EAH, and EAH + FGF4 groups. **D** Representative immunofluorescence images of F4/80 expression in liver sections from the control, EAH, and EAH + FGF4 groups. Scale bar, 20 μm. **E** Heatmap of representative differentially expressed genes related to M1-macrophage and M2-macrophage marker genes in livers from the EAH and EAH + FGF4 groups, based on RNA-seq data. **F** Changes in relative mRNA-expression levels of genes encoding M1-macrophage markers (*Nos2* and *Cd86*) and M2-macrophage markers (*Arg1* and *Cd206*) in livers of the control, EAH, and EAH + FGF4 groups. **G** Flow-cytometric analysis of liver macrophages (F4/80 + CD11b + cells), M1 macrophages (CD86 + cells), and M2 macrophages (CD206 + cells), along with their quantitative results, for the EAH and EAH + FGF4 groups. **H** WB analyses of NOS2 and ARG1 expression differences in total liver lysates from the control, EAH, and EAH + FGF4 group, as well as quantitation of the results. The WB data were quantified using ImageJ software. n = 3–6 mice/group. Statistical comparisons were made using one-way ANOVA with Tukey’s post-hoc test and the two-tailed, unpaired Student’s t-test; *p < 0.05, **p < 0.01, ns, not significant

To gain insight into the pathophysiological importance of FGF4 in the liver, we administered FGF4 to EAH mice. Treatment with FGF4 resulted in lower AST and ALT levels in EAH mice compared with that in EAH mice (Fig. 4A). FGF4-treated EAH mice showed lower liver tissue necrosis and inflammatory-cell infiltration than untreated EAH mice (Fig. 4B). Consistently, inflammatory cytokines were expressed at lower levels in FGF4-treated mice and anti-inflammatory cytokines were produced at higher levels (Fig. 4C). Furthermore, following FGF4 treatment, the abundance of F4/80 + macrophages noticeably decreased in EAH mice (Fig. 4D). RNA-seq revealed that FGF4 treatment reduced the levels of M1-macrophage markers but increased those of M2-macrophage markers (Fig. 3E). Our flow-cytometric, qRT-PCR, and WB data confirmed the FGF4 treatment decreased M1 macrophages and increased M2 macrophages, using vehicle-treated EAH mice as a reference (Fig. 3F–H).

### Macrophages are prerequisites for FGF4-mediated amelioration of liver injury in EAH mice

**Fig. 5:**
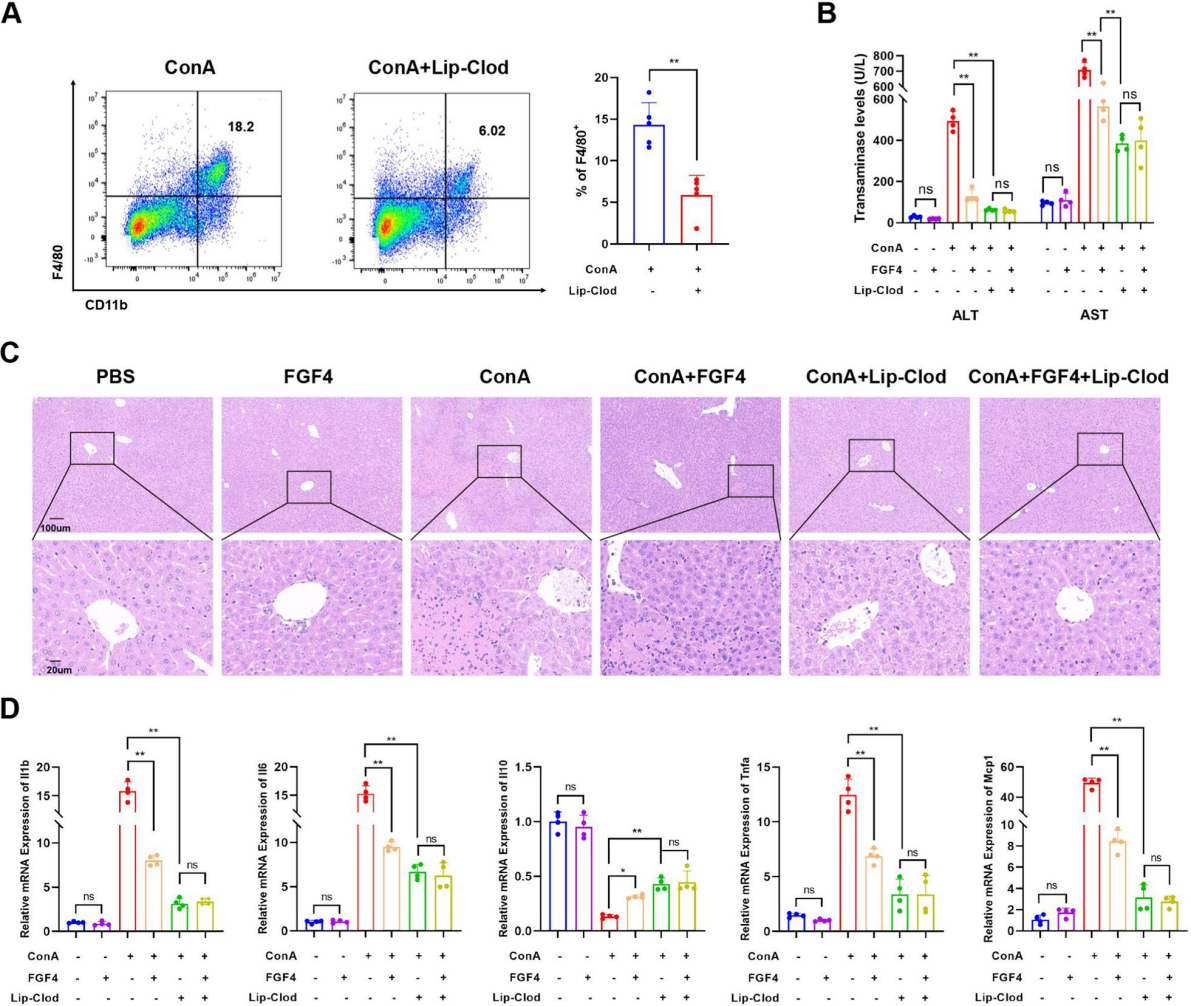
Macrophages were required for FGF4-mediated amelioration of liver injury in EAH mice. **A** Changes in flow-cytometric analysis of mouse liver macrophages (F4/80 + CD11b + cells) and their quantitative results in the EAH and EAH + clodronate liposomes (Lip-Clod)-intervention groups. **B** Serum ALT- and AST-activity levels of the indicated mouse groups. **C** Representative images of liver sections from the indicated mouse groups, stained with H&E. Scale bars, 200 μm or 50 μm. **D** Changes in relative mRNA-expression levels of inflammatory factors (*Il1b*, *Il6*, *Tnfa*, *Il10*, and *Mcp1*) in livers from the indicated mouse groups. n = 3–6 mice/group. Statistical comparisons were made using one-way ANOVA with Tukey’s post-hoc test and the two-tailed, unpaired Student’s t-test; *p < 0.05, **p < 0.01, ns, not significant

To explore the effects of FGF4 on macrophages, we depleted liver macrophages using intraperitoneal injections of clodronate liposomes. Following injection, the total macrophage counts were significantly lower in treated mice than those in untreated mice (Fig. 5A). We observed that ALT and AST levels substantially decreased after treatment with clodronate liposomes (Fig. 5B). Treatment with FGF4 significantly reduced liver tissue necrosis in EAH mice. However, combined intervention with FGF4 and clodronate liposomes similar liver tissue necrosis in EAH mice compared with that in EAH mice treated with clodronate liposomes alone (Fig. 5C). Furthermore, we evaluated the production of inflammatory factors. In EAH mice, administering FGF4 alone or clodronate liposomes with FGF4 led to reduced pro-inflammatory factor levels compared with those in the EAH group. EAH mice treated with clodronate liposomes showed no differences in the levels of secreted inflammatory factors, with or without FGF4 supplementation (Fig. 5D).

### FGF4 reduced M1-macrophage levels and prevented EAH-associated liver inflammation via PI3K activation

**Fig. 6:**
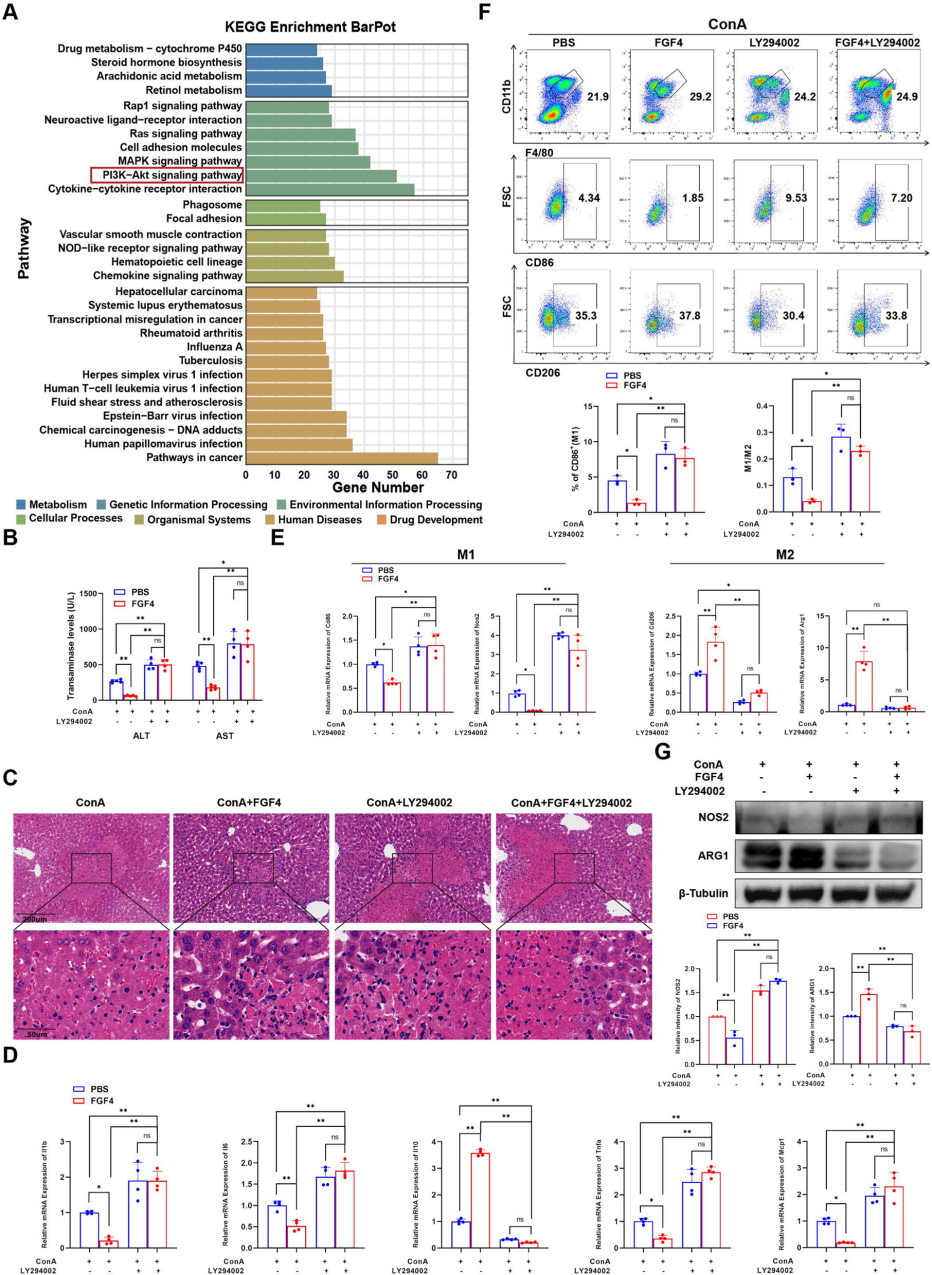
FGF4 reduced M1-macrophage abundances to prevent EAH-associated liver inflammation via PI3K activation. **A** Results of KEGG pathway-enrichment analysis in livers from the EAH and EAH + FGF4 groups based on RNA-seq data, highlighting the PI3K–AKT pathway (red box; n = 4 mice/group). **B** Serum ALT- and AST-activity levels of EAH mice treated with PBS, FGF4, a PI3K inhibitor (LY294002), or FGF4 + LY294002. **C** Representative images of liver sections from the indicated groups after H&E staining. Scale bars, 200 μm or 50 μm. **D** Changes in the relative mRNA-expression levels of inflammatory factors (*Il1b*, *Il6*, *Tnfa*, *Il10*, and *Mcp1*) in livers from mice in the indicated groups. **E** Changes in the relative mRNA-expression levels of M1-macrophage marker genes (*Nos2* and *Cd86*) and M2-macrophage marker genes (*Arg1* and *Cd206*) in livers from mice in the indicated groups. **F** Flow-cytometric analysis of changes in liver macrophage (F4/80 + CD11b + cells), M1 macrophages (CD86 + cells), and M2 macrophages (CD206 + cells), along with quantitation expression of the results. **G** WB analyses of NOS2 and ARG1 levels in total liver lysates from the indicated groups and quantitation of the results. The WB data were quantified using ImageJ software. n = 3–6 mice per group. Statistical comparisons were made using one-way ANOVA with Tukey’s post-hoc test; *p < 0.05, **p < 0.01, ns, not significant

RNA-seq analyses of the differentially expressed genes revealed the PI3K–protein kinase B (AKT)-signaling pathway as one of the most significantly altered pathways (Fig. 6A). The PI3K inhibitor, LY294002, inhibited the ability of FGF4 to diminish transaminase levels, liver tissue necrosis, F4/80 + macrophage infiltration, and inflammatory factor production (Fig. 6B–D). By conducting genetic, protein, and cellular-level analyses, we ascertained that inhibiting PI3K also inhibited the capability of FGF4 to reduce the abundance of M1 macrophages (Fig. 6E–G).

**Fig. 7:**
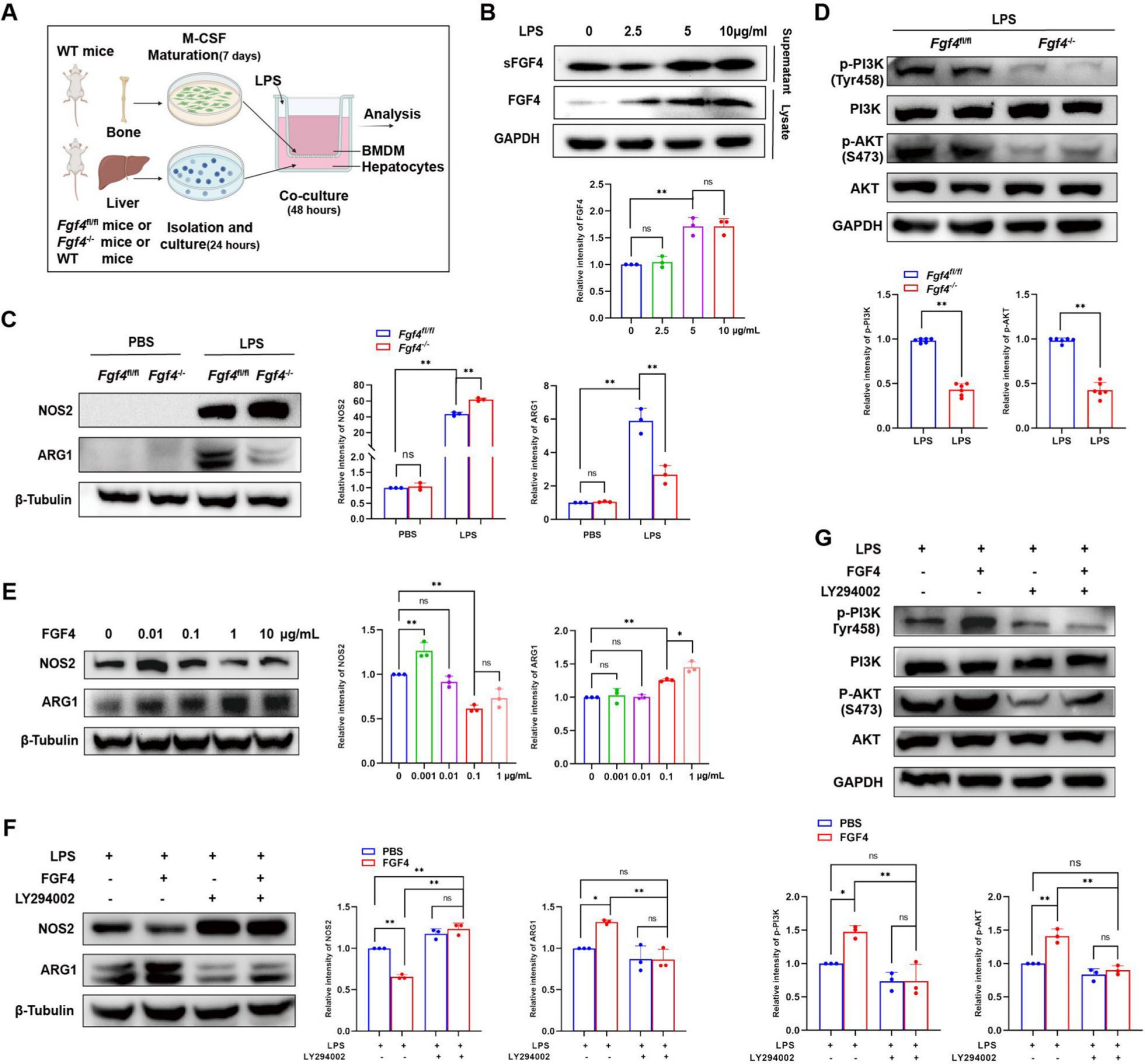
FGF4 reduced M1 macrophage abundances via the PI3K–AKT pathway in co-cultured primary hepatocytes and BMDMs. **A** Schematic representation of the experimental design for co-culturing primary BMDMs with primary hepatocytes. **B** WB analysis of FGF4 expression in primary hepatocytes and supernatants from co-cultured cells treated with PBS or LPS, along with the quantitative results. Co-cultured cells were treated with LPS at 0, 2.5, 5, or 10 μg/mL for 24 h. **C** WB analysis of NOS2 and ARG1 expression in primary BMDMs co-cultured with primary hepatocytes from *Fgf4*^fl/f^^l^ and *Fgf4*^*−/−*^ mice and the quantitative results. The co-cultured hepatocytes were treated with LPS (5 µg/mL) for 24 h. **D** WB analysis of PI3K, phospho-PI3K, AKT, and phospho-AKT protein expression and the quantitative results for the indicated groups. **E** WB analysis of NOS2 and ARG1 in BMDMs treated with PBS or FGF4, along with the quantitative results. The co-cultured cells were treated with FGF4 at 0, 0.01, 0.1, 1 or 10 μg/mL for an additional 0.5 h, followed by stimulation with LPS (5 µg/mL) for 24 h. **F** WB analysis of NOS2 and ARG1 expression in the indicated groups and the quantitative results. Co-cultured cells were pretreated with LY294002 (20 µM) or PBS for 1 h, then with FGF4 (1 μg/mL) or PBS were for an additional 0.5 h, followed by stimulation with LPS (5 µg/mL) for 24 h. **G** WB analysis of PI3K, phospho-PI3K, AKT, and phospho-AKT protein expression and quantitation of the results for the indicated groups. The WB data were quantified using ImageJ software. Statistical comparisons were made using one-way ANOVA with Tukey’s post-hoc test and two-tailed, unpaired Student’s t-test; *p < 0.05, ** *p*< 0.01, ns, not significant

**Fig. 8:**
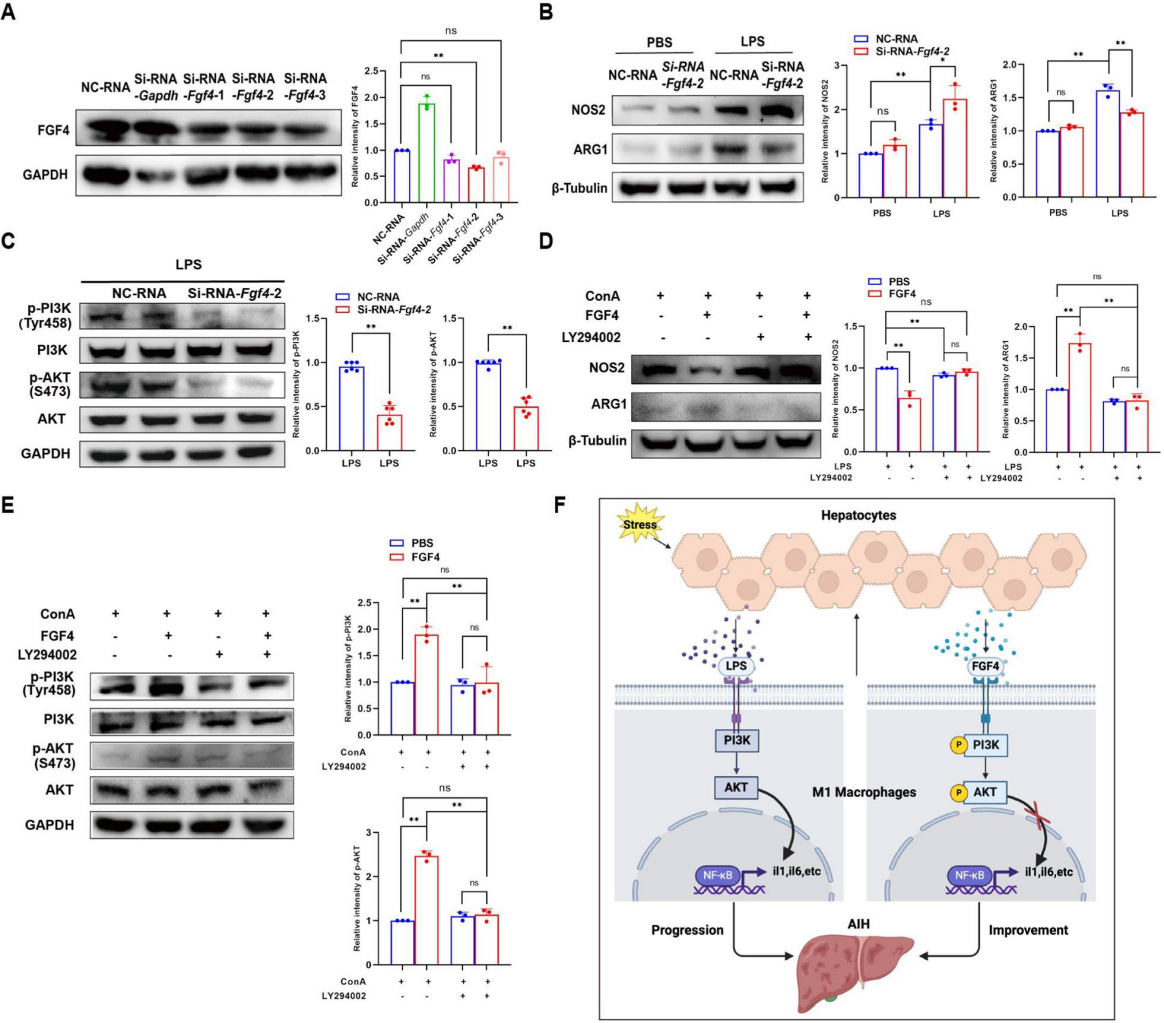
FGF4 reduced M1-macrophage abundances via the PI3K–AKT pathway in a co-culture system with AML12 cells and RAW cells. **A** Semi-quantitative WB analyses of FGF4 expression in AML12 cells transfected with control or FGF4 siRNA. **B** WB analysis of NOS2 and ARG1 expression in AML12 cells co-cultured with RAW cells transfected with a control or FGF4 siRNA, along with the quantitative results. **C** WB analysis of PI3K, phospho-PI3K, AKT, and phospho-AKT protein expression and quantitation of the results obtained with the indicated groups. **D** WB analysis of NOS2 and ARG1 expression in the indicated groups and the quantitative results. **E** WB analysis of PI3K, phospho-PI3K, AKT, and phospho-AKT protein expression and the quantitative results for the indicated groups. **F** A mechanistic illustration of the role of FGF4 in regulating liver inflammation in EAH. Autoantigens lead to liver injury and increased FGF4 secretion. FGF4 promotes the activation of PI3K–AKT signaling to reduce the abundance of M1 macrophages, which aggravates liver injury and inflammation. The WB data were quantified using ImageJ software. The data shown represent three independent experiments. Statistical comparisons were made using one-way ANOVA with Tukey’s post-hoc test and the two-tailed, unpaired Student’s t-test; *p < 0.05, **p < 0.01, ns, not significant

We also performed vitro experiments, in which primary hepatocytes were co-cultured with BMDMs (Fig. 7A). Peak FGF4 secretion from hepatocytes occurred when stimulating them with an LPS concentration of 5 µg/mL; thus, we selected that concentration for subsequent experiments (Fig. 7B). FGF4 deficiency increased NOS2 expression and reduced ARG1 expression after LPS stimulation (Fig. 7C). These results were associated with lower levels of phospho-PI3K and phospho-AKT (Fig. 7D). After knocking down *Fgf4* in AML12 cells, we discovered that FGF4 deficiency facilitated NOS2 expression and inhibited the level of phospho-PI3K and phospho-AKT expression after LPS stimulation (Fig. 8A–C).

Subsequently, we investigated alterations in macrophage polarization in response to different concentrations of FGF4 in vitro under LPS stimulation. Notably, a discernible therapeutic effect was found using a FGF4 concentration of 1 µg/mL (Fig. 7E). When compared with the LPS stimulation group, FGF4 supplementation led to reduced NOS2 expression and augmented ARG1 expression. However, in LPS stimulation groups, the therapeutic impact of combination treatment with LY294002 and FGF4 exhibited an efficacy similar to that observed following in vitro treatment with LY294002 (Fig. 7F, 8D). Furthermore, the FGF4-treated group demonstrated markedly higher phospho-PI3K and phospho-AKT production than the control group. Conversely, the group treated with LY294002 and FGF4 showed phospho-PI3K and phospho-AKT levels that were similar to those of the LY294002 group (Fig. 7G, 8E). Collectively, our findings substantiate a mechanism whereby FGF4 confers hepatic protection against inflammation by diminishing the presence of M1 macrophages through the PI3K–AKT-signaling pathway.

## Discussion

In this study, we observed that liver tissues from patients with AIH exhibited greater macrophage infiltration than those from healthy control subjects. Further analysis suggested that macrophage polarization might play a pivotal role in AIH pathogenesis and progression. We also found that the abundance of M1 macrophages was positive associated with FGF4 expression. To clarify the role of FGF4, we use liver-specific FGF4-knockout mice (*Fgf4*^−/−^) and control mice (*Fgf4*^fl/fl^) lacking the knockout. *Fgf4*^−/−^ EAH mice exhibited heightened liver tissue necrosis and more abundant M1 macrophages than the control *Fgf4*^fl/fl^ EAH mice. Pharmacological treatment with FGF4 (which lacks mitogenic activity) showed reduced liver damage in association with decreased M1 macrophages. Nonetheless, after macrophage ablation, the ameliorative impacts of FGF4 treatment waned, indicating that the macrophage population is the primary conduit for the anti-inflammatory effects of FGF4. By performing comprehensive in vivo and in vitro analyses, we conclusively established that FGF4 operates as an anti-inflammatory agent, potentially by activating the PI3K–AKT in pathway hepatic macrophages, leading to suppressed M1 macrophage polarization.

Recent findings have highlighted the crucial roles of macrophage polarization in diverse physiological and pathological processes, which encompass inflammation, tumorigenesis, tissue repair, and metabolism [24, 25]. Intriguingly, these processes are notably relevant to liver diseases, implying that macrophage polarization could play a part in the development and advancement of hepatic conditions like viral hepatitis, fatty liver disease, liver fibrosis, and hepatocellular carcinoma [26–28]. M1 macrophages are pro-inflammatory and produce inflammatory molecules, such as IL-6, TNF-α, and IL-1β. In contrast, M2 macrophages are pro-reparative and secrete anti-inflammatory substances, such as IL-10 [29, 30]. We also found elevated IL-6 and IL-10 levels in peripheral blood mononuclear cells from patients with AIH. The dominance of IL-6 over IL-10 indicated that, overall, the monocytes were in a pro-inflammatory state. Previous reports also showed elevated TNF-α and IL-10 levels in monocytes from patients with AIH, with TNF-α surpassing IL-10 levels [31], aligning with our findings. This elevation stemmed from monocyte activation during the active AIH phase, which triggered adaptive immune responses characterized by increased proliferation of and IFN-γ secretion by CD4 + and CD8 + T cells. Elevated IFN-γ levels stimulate monocyte polarization towards a pro-inflammatory state, prompting the release of cytokines such as IL-6, TNF-α, and IL-1β, which contribute to tissue damage. To counter excessive immune-driven tissue injury, the body fosters anti-inflammatory cell proliferation and the secretion of mediators like IL-10. Identifying targets that modulate hepatic macrophage polarization may thus provide a potential new treatment option for AIH. However, the in vivo-adoptive reduction of M1 macrophages faces challenges such as safety concerns and limited cell sources, making widespread clinical application challenging [32]. Hence, identifying targets for regulating macrophage polarization is particularly crucial in the context of AIH.

Apoptosis is the main way that liver cells die in AIH, often displaying self-antigens on their cell surfaces. The failure of immune cells to clear excessive apoptotic cells promptly can trigger autoimmune diseases and cause tissue damage [33]. M1 macrophages are recognized for their strong antigen presentation [33]. Our findings show that during the previous phase of EAH (0–18 h), cell apoptosis intensifies, predominantly involving M1 macrophages with antigen presentation abilities. These M1 macrophages use pro-inflammatory cytokines to eliminate harmful substances. After 24 h, the abundances of M1 macrophages and apoptotic cells decreased, and M2 macrophages became more abundant. Importantly, M2 macrophages can remove apoptotic cells and regulate the release of inflammatory cytokines.

In a study of non-alcoholic fatty liver disease, FGF4 supplementation reduced liver inflammation by inhibiting hepatocyte apoptosis and adipose macrophage infiltration [15]. In this study, we found that FGF4-expression levels correlated positive with the degree of inflammation and abundance of M1 macrophages. A dynamic trend has been observed regarding FGF4 during the EAH disease process, implying that in EAH, hepatocytes adaptively produce FGF4 in response to challenges and thereby inhibit the production of pro-inflammatory factors and promote damage healing. Consequently, FGF4 levels peak in parallel with inflammation and then begin to decrease when hepatocyte damage heals. By manipulating FGF4 levels through knockdown and supplementation, we found that the anti-inflammatory impact of FGF4 on the liver was linked to its modulation of M1 macrophages. In support of this possibility, we also found that liver macrophage depletion diminished the therapeutic effect of FGF4.

Yet, the molecular mechanisms whereby FGF4 regulates M1 macrophage polarization remain undisclosed. In this study, peak FGF4 secretion from hepatocytes occurred after stimulating them with LPS at a concentration of 5 µg/mL, suggesting that liver cell inflammation and damage occurred most severely at that concentration [34, 35] and prompted hepatocytes to secrete a large amount of FGF4 to promote cellular repair. In this research, we discovered that FGF4 may exert its effects by activating the PI3K–AKT pathway. Previous results showed that FGF4 promoted bone marrow mesenchymal stem cell proliferation by activating the PI3K–AKT-signaling pathway [36]. The PI3K–AKT pathway not only controls macrophage survival, migration, and proliferation, but it also orchestrates macrophage responses to various metabolic and inflammatory cues [37]. The PI3K–AKT pathway is essential for inhibiting proinflammatory responses and increasing anti-inflammatory responses in macrophages [38]. The activation or overexpression of PI3K or AKT inhibited LPS-induced M1 macrophage activation, but nonspecific chemical inhibition of PI3K signaling increased nuclear factor-kappa B activation and inducible NOS2 production, which increased M1 macrophage responses [39]. Those findings are consistent with the results of this study.

This study had some limitations. Primarily, ConA-induced hepatitis models cannot accurately represent the state of immunity, although it was extensively characterized with our mouse model of AIH. Stimulating hepatocytes with LPS does not accurately reflect AIH cells either. Therefore, larger clinical samples are required. In addition, other aspects of macrophage biology associated with M1/M2 polarization (such as phagocytosis, autophagy, apoptosis, and metabolism) were not investigated. To demonstrate that FGF4 exerts its anti-inflammatory effects in AIH through the PI3K–AKT pathway, it will be necessary to utilize PI3K-knockout mice for validation. With the rapid development of nanomaterials [40], it is possible to employ nanodrug-delivery systems for targeted intervention against FGF4. Thus, the abovementioned problems should be solved in future research.

## Conclusion

Our findings indicate that FGF4 is a novel factor that ameliorates liver inflammation by activating the PI3K–AKT-signaling pathway to reduce the abundance of M1 macrophages. Our findings also imply that non-mitogenic FGF4 potentially represents a new therapeutic strategy against AIH.

### Supplementary Information

**Additional file 1. **Additional materials.

## Data Availability

All data generated or analyzed during this study are included in this work and are accessible upon request from the authors.
