# Supplementary material for: FGF4 ameliorates the liver inflammation by reducing M1 macrophage polarization in experimental autoimmune hepatitis
Source: J Transl Med. 2024 Aug 2;22:717. doi: 10.1186/s12967-024-05219-2 (PMC11295337; doi:10.1186/s12967-024-05219-2)
Supplement: Supplementary file 1 — Additional file 1. Additional materials. [file 12967_2024_5219_MOESM1_ESM.docx]

**Additional file 1**

**FGF4 Ameliorates the Liver Inflammation by Reducing M1 Macrophage Polarization in Experimental Autoimmune Hepatitis**

Jing Lin^a,b#^ · Hong-wei Lin^a#^ · · Yu-xing Wang^a^ · Yan Fang^a^ · Hui-mian Jiang^a^ · Ting Li^a^ · Jia Huang^a^ · Hua-dong Zhang^a^ · Xiong Ma^c^ · Da-zhi Chen^d*^ and Yong-ping Chen^a**^

^a^ The First Affiliated Hospital of Wenzhou Medical University, Hepatology Institute of Wenzhou Medical University, Zhejiang Provincial Key Laboratory for Accurate Diagnosis and Treatment of Chronic Liver Diseases, Wenzhou, 325000 Zhejiang, China

^b^ Department of Infectious Diseases, The First Affiliated Hospital of Soochow University, Suzhou, 215006, China,

^c^ Division of Gastroenterology and Hepatology, Key Laboratory of Gastroenterology and Hepatology, Ministry of Health, State Key Laboratory for Oncogenes and Related Genes, Renji Hospital, School of Medicine, Shanghai Jiao Tong University; Shanghai Institute of Digestive Disease, Shanghai 200000, China

^d^ Department of Clinical Medicine, Hangzhou Medical College, Hangzhou, China 310053

^#^ These authors contributed equally to this work.

^*^ Corresponding author. No. 481 binwen Road, Binjiang District, Hangzhou, Zhejiang Province, China. E-mail addresses: dazhichen@126.com (DZ. Chen).

^**^ Corresponding author. Nanbaixiang, Ouhai Direct, Wenzhou City, Zhejiang Province, China

E-mail addresses: [cyp@wmu.edu.cn](mailto:cyp@wmu.edu.cn) (YP. Chen).

**Table S1–Mouse genotyping primer sequences**

| Gene | 5’ Primer | 3’ Primer |
| --- | --- | --- |
| Fgf4-5wt | GGCAGAGGCAGGCACATTTATG | GCCTCACTAAGCCACACCTGTTC |
| Fgf4-3wt | AGCATCTGGGAGGCAAAAGC | GAACAACTTTGCTGAGGAGCCC |
| Alb-Cre | GGGCAGTCTGGTACTTCCAAGCT | TAGCTACCTATGCGATCCAAACAAC |
| Internal control for Cre | CAAATGTTGCTTGTCTGGTG | GTCAGTCGAGTGCACAGTTT |

**Table S2–Primer sequences**

| Gene | 5’ Primer | 3’ Primer |
| --- | --- | --- |
| β-actin | CCTCACTGTCCACCTTCC | GGGTGTAAAACGCAGCTC |
| Mcp1 | AGGCCCAGAGCAAGAGAGGTA | CTGCTGCTGGTGATCCTCTTG |
| iNos | AATCTTGGAGCGAGTTGTGG | CAGGAAGTAGGTGAGGGCTTG |
| Arg-1 | CTCCAAGCCAAAGTCCTTAGAG | AGGAGCTGTCATTAGGGACATC |
| IL-10 | GCTCTTACTGACTGGCATGAG | CGCAGCTCTAGGAGCATGTG |
| Cd206 | CAAGGAAGGTTGGCATTTGT | CCTTTCAGTCCTTTGCAAGC |
| Cd86 | TGTTTCCGTGGAGACGCAAG | TTGAGCCTTTGTAAATGGGCA |
| TNF-α | AAGCCTGTAGCCCACGTCGTA | GGCACCACTAGTTGGTTGTCTTTG |
| IL-1β | CTGAACTCAACTGTGAAATGCCA | AAAGGTTTGGAAGCAGCCCT |
| Ym-1 | GCTCTTCATCTGTCAGCTTTGG | TGTGAGAGCAAGAAACAAGCAT |
| IL-6 | TGGAAATGAGAAAAGAGTTGTGC | TGGAAATGAGAAAAGAGTTGTGC |
| Fgfr1c | GGTGCTTCATCTACGGAATGTCTCC | TCGGAGACTCCAGCCAGCATGG |
| Fgfr2b | TAAATAGCTCCAATGCAGAAGTGC | GGTGTCCGCTGTTGAGGACAGACG |
| Fgfr2c | GGAATGTAACTTTTGAGGATGCTGG | GGTGTCCGCTGTTGAGGACAGACG |
| Fgfr3c | ACACCACCGACAAGGAGCTAGAGG | CTTGTCGATGCCAATAGCTTCTGC |
| Fgfr4 | CCTGAAGACAACAGACATCAATAGC | GTTGATGATGTTCTTGTGTCTTCCG |

**Table S3–Experimental reagents and antibodies**

| REAGENT or RESOURCE | SOURCE | IDENTIFIER | Application |
| --- | --- | --- | --- |
| **Antibodies** |  |  |  |
| liver Arginase 1/ARG1 antibody | santa cruz | sc-271430 | WB(1:100) |
| NOS2 antibody | santa cruz | sc-7271 | WB(1:100) |
| Anti-FGF4 Antibody | Abcam | ab106355 | WB(1:1000) |
| Phospho-PI3 Kinase p85 (Tyr458)/p55 (Tyr199) Antibody | Cell Signaling Technology | 4228S | WB(1:1000) |
| PI3 Kinase p85 (19H8) Rabbit mAb | Cell Signaling Technology | 4257S | WB(1:1000) |
| Phospho-Akt (Ser473) Antibody | Cell Signaling Technology | 9271S | WB(1:1000) |
| Akt Antibody | Cell Signaling Technology | 9272S | WB(1:1000) |
| GAPDH Monoclonal antibody | Proteintech | 1E6D9 | WB(1:10000) |
| HRP-labeled Goat Anti-Mouse IgG(H+L) | Epizyme | LF101 | WB(1:5000) |
| HRP-labeled Goat Anti-Rabbit IgG(H+L) | Epizyme | LF102 | WB(1:5000) |
| CD86 Rabbit pAb (A16805) | ABclonal | A16805 | IF(1:200) |
| EMR1 Rabbit pAb | ABclonal | A1256 | IF(1:200) |
| MRC1 Antibody | Affinity | DF4149 | IF(1:200) |
| Dylight 488,Donkey Anti-Rabbit IgG(H+L) | Yeasen | E032221-01 | IF(1:1000) |
| Dylight594, Goat Anti-Mouse IgG(H+L) | Yeasen | E032410 | IF(1:1000) |
| CD206 Monoclonal antibody | Proteintech | 60143-1-Ig | IHC(1:20000) |
| NOS2 antibody | santa cruz | sc-7271 | IHC(1:100) |
| EMR1 Polyclonal antibody | Proteintech | 27044-1-AP | IHC(1:800) |
| PE/Cyanine7 Rat IgG2b, κ Isotype Ctrl Antibody | Biolegend | 400617 | FCM |
| PE/Cyanine7 anti-mouse/human CD11b Antibody | Biolegend | 101216 | FCM |
| Brilliant Violet 421™ anti-mouse F4/80 Antibody | Biolegend | 123131 | FCM |
| APC anti-mouse CD206 (MMR) Antibody | Biolegend | 141708 | FCM |
| PE Rat IgG2a, κ Isotype Ctrl Antibody | Biolegend | 400507 | FCM |
| TruStain FcX™ (anti-mouse CD16/32) Antibody | Biolegend | 101320 | FCM |
| APC Rat IgG2a, κ Isotype Ctrl Antibody | Biolegend | 400511 | FCM |
| PE anti-mouse CD86 Antibody | Biolegend | 105008 | FCM |
| FITC Rat IgG2b, κ Isotype Ctrl Antibody | Biolegend | 400633 | FCM |
| Brilliant Violet 421™ Rat IgG2a, κ Isotype Ctrl Antibody | Biolegend | 400535 | FCM |
| FITC anti-mouse CD45 Antibody | Biolegend | 103108 | FCM |
| Brilliant Violet 421™ anti-mouse F4/80 | Biolegend | 123137 | FCM |
| Fixable Viability Stain 510 | BD Bioscience | 564406 | FCM |
| **Chemicals, Peptides, and Recombinant proteins** |  |  |  |
| Concanavalin A(ConA) | Sigma-Aldrich | slcg9227 |  |
| Clodronate Liposomes and Control Liposomes | Liposoma | CP-005-05 |  |
| LY29004 | MCE | L9908 |  |
| Collagenase IV | Sigma-Aldrich | C5138 |  |
| DNase I | Sigma-Aldrich | DN25 |  |
| DMEM basic（1X） | Gibco | C11995500BT |  |
| DMEM/F-12（1:1）basic（1X） | Gibco | C11330500BT |  |
| Percoll® | Sigma-Aldrich | P1644 |  |
| Hanks | Solarbio | H1020 |  |
| D-Hanks | Solarbio | H1045 |  |
| Flow cytometry Staining buffer | MultiSciences | 70-S1001 |  |
| Fix&Perm Kit | MultiSciences | 70-GAS006/2 |  |
| Phosphate Buffered Saline (PBS) | solarbio | P1020 |  |
| Fetal Serum Bovine(FBS) | Gibco | 10099-141 |  |
| Penicillin-Streptomycin, Liquid | Gibco | 15140122 |  |
| EDTA | Gibco | 2520007 |  |
| Lipofectamine™ 2000 Transfection Reagent | Thermo Fisher Scientific | 11668030 |  |
| Opti-MEM Reduced Serum Medium | Thermo Fisher Scientific | 31985-070 |  |
| Recombinant Murine GM-CSF | peprotech | 315-03 |  |
| RBC Lysis Buffer(Multi-species) | solarbio | R1010 |  |
| Lipopolysaccharides from Escherichia coli 055:B5 | Sigma | L2880-10MG |  |
| William's E | Procell | PM151221 |  |
| L-Glutamine，Liquid(200mM) | solarbio | G0200 |  |
| Insulin (dry powder) | Gibco | 91077C |  |
| dexamethasone | Gibco | CM4000 |  |
| cell culture chamber | NEST | 14141 |  |
| TRIzol | Thermo Fisher Scientific | 15596026 |  |
| PrimeScript RT Master Mix | Takara | RR036A |  |
| TB GreenTM Premix Ex TaqTM II(Tli RNaseH Plus) | Takara | RR820A |  |
| WesternBright Sirius Chemiluminescent Detection Kit |  | K-12043-D10 |  |
| Bovine serum albumin(BSA) | Sigma-Aldrich | B2064 |  |
| DAPI | Solarbio | S2110 |  |
| TUNEL System kit | Promega | G3250 |  |
| BCA Protein Assay Kit | Beyotime Biotechnology | P0010 |  |
| Mammalian tissue protein extraction reagent | BOSTER | AR0101 |  |
| Total protein extraction reagent for cultured cells | BOSTER | AR0103 |  |
| phosphatase inhibitors | Applygen | P1260 |  |
| PMSF | solarbio | P0100 |  |
| BeyoGel™ Plus PAGE(Tris-Gly, 10%, 15) | Beyotime Biotechnology | P0456M |  |
| PageRuler Prestained Protein Ladder | Thermo Fisher Scientific | PC26616 |  |
| **Software** |  |  |  |
| GraphPad Prism 8 | GraphPad | <https://www.graphpad.com/> | |
| Image J, V 1.44p | National Institutes of Health (NIH) | <https://imagej.nih.gov/ij/> | |
| Flow Jo, V 10 | FlowJo, LLC | <https://www.flowjo.com/> | |

**Table S4–Primer sequences for FGF4 SiRNA**

| Gene | 5’ Primer | 3’ Primer |
| --- | --- | --- |
| FGF4 SiRNA-1 | GACCCUUAGACAACUUUAUTT | AUAAAGUUGUCUAAGGGUCTT |
| FGF4 SiRNA-2 | CGACGAGUGUAAAUUCAAATT | UUUGAAUUUACACUCGUCGTT |
| FGF4 SiRNA-3 | CUACCAUGAAGGUAACCCATT | UGGGUUACCUUCAUGGUAGTT |

**2 Materials and methods**

**2.1 Histological analysis**
Liver and small intestine tissues were fixed in 4% paraformaldehyde, followed by embedding in paraffin. Subsequently, 5μm sections were obtained and subjected to hematoxylin and eosin (H&E) staining. The levels of lymphocytic infiltration, inflammatory necrosis, and disruption of liver structures were evaluated using light microscopy (Olympus, Japan).

**2.2 Biochemical Analysis and Enzyme-Linked Immunosorbent Assay (ELISA)**

Serum levels of alanine aminotransferase (ALT) and aspartate aminotransferase (AST) were determined using the AU5800 automatic biochemistry analyzer from Beckman Coulter, USA, at the clinical biochemical laboratory of the First Affiliated Hospital of Wenzhou Medical University.

**2.3 Quantitative Real-time PCR (qRT-PCR)**

mRNA levels were assessed through quantitative real-time PCR, employing SYBR Green and the primers detailed in Supplementary Table 2, as previously outlined[12]. Beta-actin served as the internal reference, and the fold changes were computed utilizing the relative quantification method (2-ΔΔCt).

**2.4 Western Blotting**

Proteins extracted from tissues were subjected to incubation with primary antibodies targeting GAPDH (A6130, Proteintech, Wuhan, China), NOS2 (sc-7271, Santa Cruz Biotechnology, Dallas, TX), ARG1 (sc-271430, Santa Cruz), p-FGFR1(ab59194, Abcam, Cambridge, UK), PI3K(4257S, Cell Signaling Technology, Inc., Boston, US), p-PI3K (4228S, CST), AKT(4691S, CST), p-AKT (9271S, CST), and FGF4 (ab106355, Abcam). Horseradish peroxidase (HRP)-labeled goat anti-rabbit polyclonal IgG antibodies (LF101 and LF102, respectively, Epizyme, Shanghai, China) were utilized as secondary antibodies (Supplementary Table 3). The relative protein levels, represented by the ratio of the gray value of the target gene to that of the internal reference, were determined with GAPDH or β-Tubulin serving as an internal control. Quantification of proteins was performed using ImageJ software (version 1.44p, NIH).

**2.5 Immunofluorescence (IF)**

Mice's liver tissues or cells were fixed with 4% paraformaldehyde. After blocking with 5% BSA for 30 min, the sections were incubated with CD206(DF4149, Affinity), CD86 (A16805, ABclonal) or F4/80 (A1256, ABclonal) overnight at 4°C. Following PBS wash, sections were incubated with secondary antibodies (E032221 and E032410 respectively, Yeasen, Shanghai, China). Fluorescence pictures were acquired under a fluorescence microscope ECLIPSE C1 (Nikon, Japan).

**2.6 Immunohistochemistry (IHC)**

The liver tissue slides were put in 5% BSA and incubated overnight at 4°C with CD206 (60143-1-Ig, Proteintech), NOS2 (sc-7271, santa cruz) or F4/80 (27044-1-AP, Proteintech). The sections were incubated with secondary antibodies that were appropriately biotinylated, stained with diaminobenzidine (DAB), and then counterstained with hematoxylin. Images obtained by optical microscopy (Olympus, Japan).

**2.7 Terminal deoxynucleotidyl transferase-mediated dUTP Nick-End Labeling (TUNEL)**

Paraffin-embedded liver sections were subjected to de-paraffinization and then stained with the DeadEnd™ Fluorometric TUNEL System TB235 kit (G3250, Promega, Southampton, UK) according to the manufacturer’s instruction. Stained liver tissues were visualized with a Nikon C2si Confocal Microscope

**2.8 Cytometric bead array (CBA)**

Peripheral plasma samples from AIH patients and healthy controls measured for cytokines using the Human Th1/Th2 Cytokine Cytometric Bead Array (CBA) Kit (20180030, cell-genebio, jiangxi, China) according to the manufacturer’s instruction. A FACSCanto II was used for CBA (BD Bioscience).
